# Epidemiologic, Entomologic, and Virologic Findings during Reemergence of Western Equine Encephalitis Virus, Argentina

**DOI:** 10.3201/eid3208.250829

**Published:** 2026-08

**Authors:** Lorena Spinsanti, Cintia Fabbri, Ana Piskorz, Victoria Luppo, Gonzalo Castro, Adrián Farías, Mauricio Beranek, Gabriel Barco, Mariel Feroci, Cintia Barulli, Jorge García, Anabel Sinchi, Anabella Fantilli, María Belén Pisano, Agostina Pierdomenico, Andrea Marcos, Rodrigo Balzano, Facundo Cuba, Sol Haim, Florencia Mallou, Carolina Paladino, Tomás Poklepovich, Liliana Luque, Brenda Konigheim, Juan Javier Aguilar, Jimena Renga, Maria Alejandra Morales, Adrián Díaz

**Affiliations:** National University of Córdoba, Córdoba, Argentina (L. Spinsanti, G. Castro, A. Farías, M. Beranek, G. Barco, A. Fantilli, M.B. Pisano, B. Konigheim, J.J. Aguilar, A. Díaz); National Administration of Laboratories and Health Institutes, Buenos Aires, Argentina (C. Fabbri, V. Luppo, M. Feroci, C. Barulli, J. García, A. Sinchi, F. Cuba, S. Haim, F. Mallou, C. Paladino, T. Poklepovich, M.A. Morales); National Service of Agrifood Health and Quality (SENASA), Buenos Aires (A. Piskorz, A. Pierdomenico, A. Marcos, R. Balzano, J. Renga); Ministry of Health of Córdoba Province, Córdoba (G. Castro, L. Luque); National Scientific and Technical Research Council (CONICET), Buenos Aires (G. Barco, M.B. Pisano, B. Konigheim, A. Diaz)

**Keywords:** western equine encephalitis virus, viruses, vector-borne infections, zoonoses, alphavirus, reemergence, epizootic, horse, mosquitoes, Argentina

## Abstract

During November 2023–April 2024, a total of 1,543 horses with neurologic disease from rural areas in 17 provinces of Argentina were reported to national animal health authorities. Nested reverse transcription PCR, quantitative real-time reverse transcription PCR, and sequencing of equine brain necropsies confirmed western equine encephalitis virus (WEEV) infection in 23 horses and 1 sheep. Phylogenetic analyses identified a lineage previously detected in Argentina in 1957, overlapping areas affected during the 1980s epizootics. Overall, we aspirated 1,362 female mosquitoes (12 species) from 1 of the most affected areas; *Aedes (Ochlerotatus) albifasciatus* (70.1%) and *Ae.* (*Ochlerotatus) scapularis* (26.8%) mosquitoes were the most abundant species. Three of 27 mosquito pools (n = 1,089 mosquitoes) were identified as WEEV positive (2 *Ae. albifasciatus*, 1 *Ae. scapularis*). Increased rainfall, agricultural expansion, vector proliferation, and low vaccination coverage were likely key factors contributing to the reemergence of WEEV.

Western equine encephalitis virus (WEEV) (species *Alphavirus western*) was first isolated in 1930 on the West Coast of the United States (*1*). The virus caused equine epizootics and human epidemics in the western, central, and southern United States. WEEV is pathogenic to humans and equids, causing a febrile illness that can progress to severe neurologic disease and death (*2*).

WEEV activity has been reported across North America (Canada, Mexico, and the United States), Central America, and South America (Argentina, Brazil, Guyana, Paraguay, and Uruguay). Its maintenance cycle is well characterized in North America and involves passerine birds (e.g., house sparrows [*Passer domesticus*] and house finches [*Hemorhous mexicanus*]) as enzootic hosts and *Culex tarsalis* mosquitoes as the primary enzootic vector. WEEV can also infect various mammals, initiating an epizootic mammal–mosquito cycle (*2*).

During 1996–2013, enzootic and epizootic WEEV activity decreased drastically in the Americas. For example, surveillance data from California showed a decline in WEEV-positive mosquito pools and seropositive birds (*3*). Both human and equine cases have declined substantially over time. In North America, the last reported human case occurred in 1999; however, a single fatal case was documented in Uruguay in 2009 in an otherwise healthy 14-year-old boy (*4*,*5*). Equine cases followed a similar trend; the last major outbreak was reported in 1975, and only sporadic, smaller outbreaks continued into the 1990s (*6*,*7*).

In Argentina, WEEV was first isolated in 1933 from a sick equine in Monte Veloz, Buenos Aires Province (MV strain) (*8*). Subsequently, several epizootics of varying magnitudes were reported in the temperate region of the country (Buenos Aires, Córdoba, Santa Fe, Entre Ríos, and La Pampa Provinces) during 1957–1958, 1972–1973, 1982–1983, and 1988–1989 (*8*,*9*). A few human cases were reported only in the southern part of the epizootic area, in Viedma, Río Negro Province (*9*). Despite the historical effects of this virus in Argentina, its ecology remains only partially characterized. WEEV strains have been isolated from various mosquito species, including *Aedes* (*Ochlerotatus*) *albifasciatus*, *Anopheles albitarsis*, *Mansonia* spp., and *Psorophora pallescens* during epizootic periods, as well as from *Culex ocossa* mosquitoes during enzootic periods (*10*,*11*). On the basis of vector competence assays, the *Ae. albifasciatus* mosquito has been implicated as an epizootic vector based on its ability to transmit the virus in laboratory experiments (*12*).

After 35 years without reported activity in Argentina, a WEEV epizootic in equines was officially reported on November 20, 2023 (*13*). The outbreak subsequently expanded to neighboring countries, including >3 laboratory-confirmed equine cases in Brazil and ≈1,000 equid cases reported in Uruguay (*14*,*15*).

From the initiation of enhanced epidemiologic surveillance in epidemiologic week (EW) 48 of 2023 through EW 31 of 2024, a total of 586 suspected human WEEV infections were notified to the National Health Surveillance System across 21 provinces in Argentina; 108 were laboratory confirmed, including several fatal cases (*16*).

A key contributing factor to the reemergence of WEEV might have been the discontinuation of mandatory vaccination. In Argentina, vaccination against equine encephalomyelitis—targeting eastern equine encephalitis virus (EEEV) and WEEV—was compulsory for all equids >3 months of age until 2016. That year, in the absence of reported cases and because domestic vaccine production was insufficient to meet national demand, mandatory vaccination was suspended, and immunization became voluntary (*17*). Vaccination has remained particularly relevant for sport and performance horses, which frequently travel long distances, including international transport by air, for competition and breeding activities.

Vaccines currently available in Argentina include 2 bivalent inactivated formulations containing EEEV and WEEV (Tecnovax, https://tecnovax.com; Instituto Rosenbusch, https://rosenbusch.com) and 1 polyvalent inactivated vaccine (Zoetis Animal Health, https://www.zoetis.com). Venezuelan equine encephalitis virus (VEEV) is considered exotic in Argentina, and vaccines containing VEEV are not licensed in the country. After the WEEV outbreak and the declaration of an animal health emergency by National Animal Health and Agrifood Quality Service (SENASA) through Resolution 1219/2023, mandatory vaccination against EEEV and WEEV was reinstated in January 2024 for all equids >2 months of age. In this study, we report the epidemiologic, virologic, entomologic, and molecular findings associated with the 2023–2024 WEEV epizootic in Argentina, providing an integrated characterization of the outbreak.

## Materials and Methods

### Sample Collection

During November 2023–April 2024 (late spring through early autumn), cases of neurologic disease in equines were reported to SENASA by private veterinarians and laboratories across 17 provinces in Argentina. Epidemiologic data, such as the number of clinical cases at each premise, vaccination status, sex and age of affected animals, and clinical manifestations, were obtained by SENASA from each notification. Each premise’s notification was considered an outbreak of neurologic disease, and laboratory confirmation of the etiology was initially assessed for each reported case. Twenty days after the index case, laboratory confirmation was performed only for outbreaks occurring in geographic areas with no previously confirmed WEEV diagnosis; suspected cases were considered positive by clinical and epidemiologic criteria in provinces that already had >1 laboratory-confirmed WEEV case.

SENASA analyzed the EW, demographic data, and number of affected equids in each outbreak on the basis of notification dates, location of affected premises, and specific epidemiologic data. After notification, central nervous system (CNS) samples (including brain, cerebellum, and spinal cord) were collected through necropsy from 49 animals (47 equines and 2 sheep) exhibiting neurologic signs compatible with CNS disease. Because veterinarians collected samples at various outbreak sites, the exact interval between death and sampling could not always be determined. Depending on local logistical capacity and the availability of dry ice, some samples were transported refrigerated at 4°C to SENASA, where they were stored at −70°C upon arrival. Samples were subsequently shipped on dry ice to the Arbovirus Laboratory at the “Dr. J.M. Vanella” Institute of Virology (InViV), National University of Córdoba (Córdoba Province), and the Arbovirus Laboratory at the National Institute of Human Viral Diseases (INEVH), Ministry of Health of Argentina (Buenos Aires Province). To ensure swift reception and processing, samples were routed to either INEVH or InViV on the basis of the geographic location of the outbreaks.

### Molecular Detection of WEEV

At InViV, we prepared 10% (wt/vol) homogenates by placing 1 g of tissue in 9 mL of minimal essential medium (MEM) supplemented with fetal bovine serum (FBS) and antibiotics and ground it using a sterilized mortar and pestle. We clarified tissue homogenates by centrifugation at 2,500 × *g* for 10 minutes at 4°C. We performed nucleic acid extraction on 140 μL of the supernatant from each sample using the High Pure PCR Template Preparation Kit (Roche, https://www.roche.com) according to the manufacturer’s instructions. We eluted extracted RNA in a final volume of 60 μL. At INEVH, we extracted total RNA from 50–100 mg of tissue using TRIZOL Reagent (Thermo Fisher Scientific, https://www.thermofisher.com), according to the manufacturer’s instructions, to obtain RNA in nuclease-free water.

For molecular detection, we used a nested reverse transcription nested PCR (RT-PCR) for the generic detection of Alphaviruses (*18*), which amplifies a 195-bp genomic fragment of the nonstructural protein 4 (nsP4) gene. Once WEEV was suspected, we confirmed the diagnosis using a WEEV-specific quantitative reverse transcription PCR (qRT-PCR) according to a previously established protocol (*19*). For all PCRs, we used supernatants from viral stocks of VEEV (TC83 strain), Madariaga virus (Cba55 strain), and WEEV (AG80–646 strain) as positive controls and sterile water as a negative control. In addition, we tested all negative samples for West Nile virus and St. Louis encephalitis virus following previously published protocols (*20*,*21*)

### Partial Genome Sequencing

We subjected amplified products from the first round of the nested RT-PCR to sequencing. We purified the nsP4 PCR products using the ExoSAP-IT PCR Product Cleanup Reagent (Thermo Fisher Scientific) or the QIAquick PCR Purification Kit (QIAGEN, https://www.qiagen.com), following the manufacturers’ instructions. We sequenced purified products using the Sanger method using the BigDye Terminator version 3.1 Cycle Sequencing Kit and the BigDye XTerminator Purification Kit (Thermo Fisher Scientific).

### Viral Isolation and Whole-Genome Sequencing

We attempted viral isolation from samples that tested positive for WEEV by RT-PCR or qRT-PCR. In brief we inoculated a 500-μL aliquot of 10% equine CNS homogenate onto Vero cells (African green monkey kidney; CRL-1586, ATCC), as previously described (*22*). We allowed the inoculum to absorb for 1 hour at 37°C in a controlled 5% CO2 atmosphere. After absorption, we washed monolayers twice with MEM and added 5 mL of fresh MEM containing 2% FBS and antibiotics. We incubated inoculated cell cultures at 37°C with 5% CO2 and examined them daily for cytopathic effect (CPE) for 14 days. We harvested infected cells showing CPE and evaluated them by RT-PCR or qRT-PCR. We titrated virus isolates in Vero cells and calculated viral titers as PFUs per milliliter (*23*).

For whole-genome sequencing of isolated strains, we extracted viral RNA from the supernatant of infected cell cultures using the MagNA Pure 96 DNA and Viral NA Large volume Kit on the MagNA Pure 96 platform (Roche) or the QIAamp Viral RNA Mini Kit (QIAGEN). For the strain isolated at InViV, library preparation was performed at the Bioinformatics and Genomics Unit, Malbrán Institute, Ministry of Health of Argentina, using the Illumina RNA Prep with Enrichment (L) Tagmentation Kit (https://www.illumina.com). That process included reverse transcription, second-strand DNA synthesis, and tagmentation using EBLTL beads with tagmentases. Subsequently, we used the Illumina Viral Surveillance Panel with specific probes to sequence the complete genomes. We amplified the hybridization product by PCR and quantified with a Qubit 3.0 fluorometer (Thermo Fisher Scientific) using the Qubit dsDNA HS Assay Kit (Invitrogen). We performed sequencing on a NovaSeq 6000 instrument (Illumina) using paired-end reads (2 × 150 bp). At INEVH, we converted extracted RNA to cDNA and prepared libraries using the Illumina COVIDSeq Assay kit—replacing SARS-CoV-2–specific primers with random hexamers—to simulate a metagenomic sequencing strategy. We performed sequencing on a MiSeq instrument (Illumina) using the MiSeq v2 reagent kit with a 300-cycle program.

### Genome Assembly and Phylogenetic Analyses

Sequences obtained using the Viral Surveillance Panel underwent standard quality control. We used FastQC (http://www.bioinformatics.babraham.ac.uk/projects/fastqc) to assess sequence quality and used Trim Galore for quality trimming using a Q30 threshold. We mapped reads to the WEEV reference sequence showing the highest percentage of identity and coverage (GenBank accession no. GQ287640). We generated a consensus genome using Snippy (https://github.com/tseemann/snippy). We screened sequence reads obtained at INEVH for quality and assembled them into complete consensus sequences using Genome Detective software version 2.14.1 (https://www.genomedetective.com).

To investigate the phylogenetic relationships of WEEV strains identified in Argentina during this epizootic, we constructed 2 datasets: a dataset of partial 440 pb nsP4 fragment sequences obtained during this study and a dataset consisting of the 3 whole-genome sequences obtained during this study, designated WEEV reference sequences, and sequences representing all previously described WEEV lineages (2*4*) (Appendix). We aligned and sequenced all sequences using MAFFT (*25*). We inferred 2 maximum-likelihood phylogenetic trees using IQ-TREE version 2 (*26*) under a general time-reversible plus empirical base frequencies plus discrete Gamma model with 4 rate categories (F plus G4) model for whole-genome sequencing and a Tamura-Nei model with equal base frequencies (TNe) plus G4 model for the nsP4 partial fragment, as determined by ModelFinder (*27*). We used the ultrafast bootstrap approach with 10,000 replicates to determine statistical support for nodes (*28*).

### Mosquito Collection and Molecular Detection of Alphaviruses

**Figure 1:**
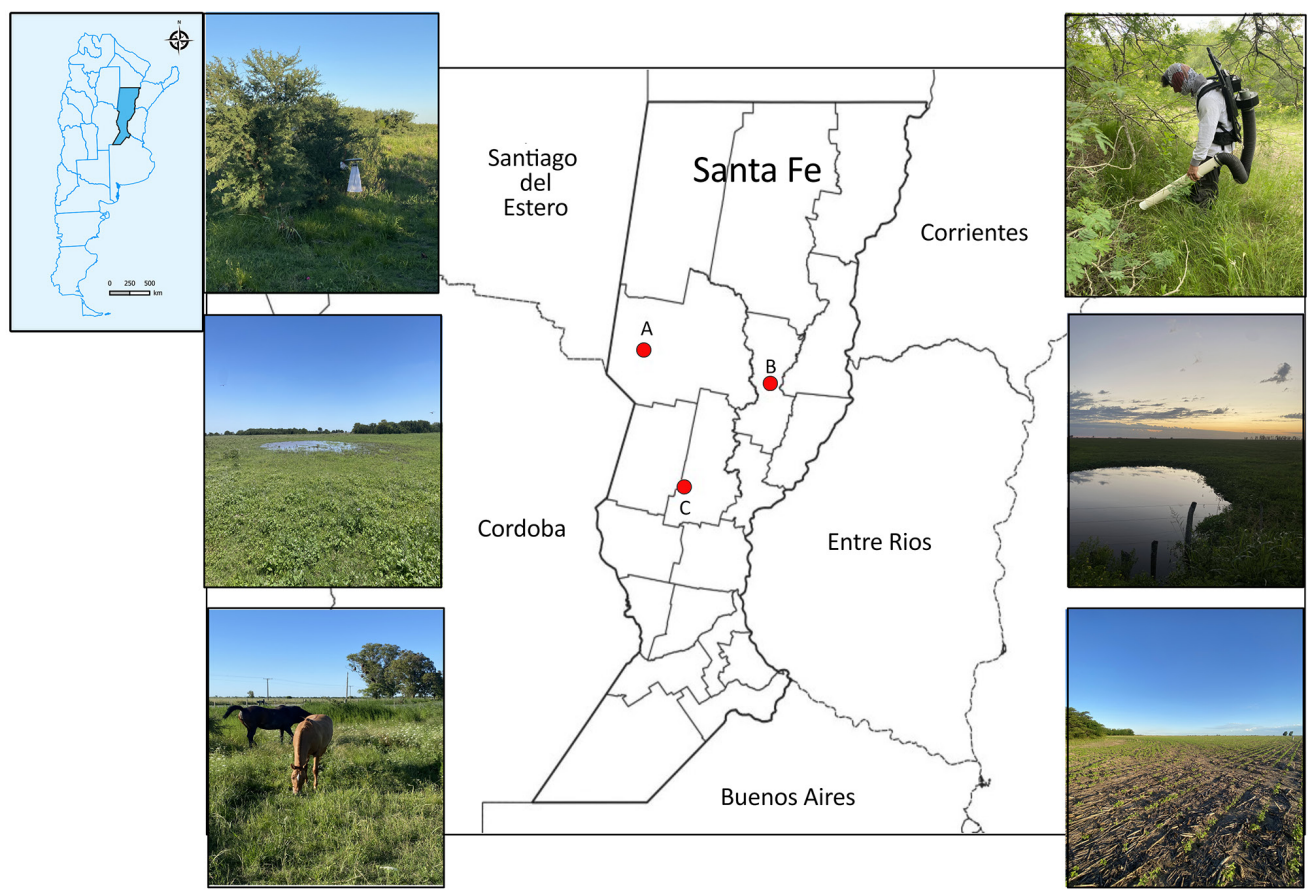
Geographic locations of mosquito collection sites in Santa Fe Province, Argentina, from study of epidemiologic, entomologic, and virologic findings during reemergence of western equine encephalitis virus, Argentina, 2023–2024. Red dots indicate collection localities: A) Arrufo, San Cristóbal Department; B) Marcelino Escalada, San Justo Department; C) Sa Pereyra, Las Colonias Department. Images are from specific sampling environments. Lefthand side from top to bottom: Centers for Disease Control and Prevention light trap in a patch of Espinal forest, temporary water pool in Arrufo, crop fields near Marcelino Escalada. Righthand side from top to bottom: mosquito aspiration in an Espinal forest patch, floodplain pool, and crop fields near Marcelino Escalada. Inset map shows location of study area in Argentina.

We collected adult mosquitoes using Centers for Disease Control and Prevention light traps baited with dry ice and backpack mechanical aspirators. Fieldwork was conducted during December 8–11, 2023, in Santa Fe Province, one of the areas most affected by the outbreak. Sampled environments consisted of Espinal forest (Sa Pereyra, Arrufó, Marcelino Escalada), crop fields (Arrufó, Sa Pereyra, Marcelino Escalada), and wetlands (lakes, ponds) (Marcelino Escalada) (Figure 1). We deployed 6 light traps per site per night in representative habitats, operating from 7:00 PM to 8:00 AM. We performed aspirator collections in the same environments during the morning (8:00 AM–12:00 PM). We placed collected mosquitoes in plastic conical tubes and transported them on dry ice to the InViV Arbovirus Laboratory. We identified specimens to the species level on a chill table using standard dichotomous keys (*29*). We pooled mosquitoes by species, collection site, habitat type, and feeding status (engorged or nonengorged). We homogenized pools in MEM supplemented with FBS and clarified them by centrifugation at 11,180 × *g* for 30 minutes at 4°C. We extracted viral nucleic acids from supernatants using the High Pure PCR Template Preparation Kit (Roche) and detected alphavirus RNA using a generic nested RT- PCR (*18*). We confirmed positive amplicons by Sanger sequencing and included the resulting sequences in the nsP4 partial sequence phylogenetic analysis.

## Results

### Epidemiologic Findings and Clinical Disease

**Figure 2:**
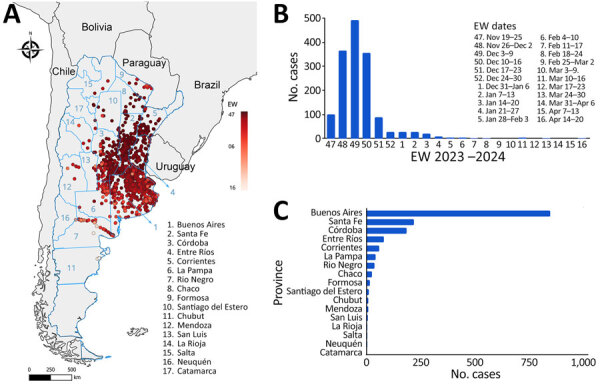
Spatiotemporal distribution of cases in study of epidemiologic, entomologic, and virologic findings during reemergence of western equine encephalitis virus, Argentina, 2023–2024. A) Spatial distribution of cases by EW. The color gradient indicates the progression timeline, ranging from earliest (dark red) to most recent (light red) cases. B) Number of reported cases per epidemiologic week. C) Geographic distribution of cases by province. EW, epidemiologic week.

During November 2023–April 2024 (EW 47 of 2023 through EW 16 of 2024), a total of 1,543 cases of WEEV-associated neurologic disease in equines were reported (Figure 2). The most affected areas were the central and northeast regions of Argentina, which have a temperate climate (Figure 2). The highest densities of equine populations are found in Buenos Aires, Santa Fe, Córdoba, Entre Ríos, and Corrientes Provinces (Figure 2). Most cases occurred in rural areas, and the affected equines had not been vaccinated against WEEV or EEEV.

Equine WEEV case reports included both laboratory-confirmed and suspected cases. Suspected cases were defined by the presence of neurologic signs and an epidemiologic link. Report notifications peaked during EW 49 (December 3–9, 2023), reaching 491 reports, and then declined to <30 cases per week from EW 52 of 2023 (December 24–30, 2023) onward. The downward trend continued until EW 16 of 2024 (April 14–20, 2024), when only 1 case was recorded in Chubut Province (Figure 2, panels B, C).

Clinical manifestations in infected equines consisted of depression or hyperexcitability; posterior, anterior, or four-limb incoordination; walking in circles; fasciculations; bruxism; hypersalivation; and recumbency within 48–72 hours. In some cases, additional signs such as jaundice, hyperthermia, nasal discharge, penile paralysis, bladder and rectal hypotonia, and loss of perineal sensibility were observed. We could not fully assess rates of illness or death because outbreaks were reported at the premise level rather than for individual animals. Most affected equids died as part of the natural course of the disease, typically within hours to 1 day after the onset of clinical signs. However, 2 equids were euthanized because of the severity of neurologic sequelae. The decline in equine cases coincided with the mandatory reinstatement of vaccination against both EEEV and WEEV in the country, suggesting it potentially affected outbreak control.

### Virus Isolation

We successfully isolated 3 WEEV strains. CPE was evident during the first passage at 48 hours postinoculation. We determined positive results by the presence of CPE and subsequently confirmed by using the WEEV-specific qRT-PCR on nucleic acids extracted from tissue culture supernatants. We harvested positive cell cultures and clarified by centrifugation under refrigerated conditions (Table 1). 

**Table 1:** Virologic and epidemiologic characteristics of equine cases in study of reemergence of western equine encephalitis virus, Argentina, 2023–2024*

Case ID	Species	Province	Location	Isolate	Viral titer, PFU/mL	GenBank accession no.†
20854_50019	Ovine	Buenos Aires	Baradero	ND	ND	PV256238
E/5193/23_18433	Equine	Buenos Aires	Capilla del Señor	ND	ND	PV256244
20788_18438	Equine	Buenos Aires	General Lavalle	ND	ND	PV256235
20793_18437	Equine	Buenos Aires	General Madariaga	ND	ND	PV256237
21251	Equine	Catamarca	Recreo	ND	ND	PV126002
20337	Equine	Chaco	Fray Justo Santa María de Oro	ND	ND	PV126010
20297	Equine	Córdoba	San Francisco	ND	ND	PV126004
20257	Equine	Corrientes	Esquina	Positive	1.3 × 10^6^	PV126003, PV391940
E5191_23_18432	Equine	Corrientes	Lavalle	Positive	1.8 × 10^7^	PV256243, PV388336
20333	Equine	Formosa	Pirane	ND	ND	PV126005
20813	Equine	La Pampa	Quemú Quemú	ND	ND	ND
21264_50024	Equine	La Rioja	General Ocampo	ND	ND	PV256240
21263	Equine	La Rioja	General San Martín	ND	ND	ND
21265_50025	Equine	La Rioja	Rosario Vera Peñaloza	ND	ND	PV256241
21312_50030	Equine	Neuquén	Senillosa	ND	ND	PV256242
20789_18435	Equine	Rio Negro	Adolfo Alsina	Positive	2.2 × 10^7^	PV256236, PV388337
20841	Equine	Salta	Rosario de la Frontera	ND	ND	PV126007
21247_50022	Equine	San Luis	General Pedernera	ND	ND	PV256239
1242–1	Equine	Santa Fe	San Cristóbal	ND	ND	PV126008
1241–4	Equine	Santa Fe	San Cristóbal	ND	ND	PV126009
1242–7	Equine	Santa Fe	San Cristóbal	ND	ND	ND
1246	Equine	Santa Fe	San Cristóbal	ND	ND	ND
20621	Equine	Santiago del Estero	Bandera	ND	ND	PV126006

*ID, identification; ND, not determined. †Corresponds to those generated in this study.

### Molecular Characterization and Phylogenetic Analyses

Of the 1,543 cases of neurologic disease, we performed laboratory diagnostics on samples from 49 animals (47 equids and 2 sheep). Among the equine samples analyzed, 48.9% (23/47) tested positive for WEEV by qRT-PCR. Of the 2 sheep sampled, 1 tested positive for WEEV (Table 1). No samples tested positive for St. Louis encephalitis virus, West Nile virus, EEEV, or VEEV. In addition, 5 WEEV-negative equine samples from animals showing neurologic signs were submitted from a rabies-endemic area; 2 (40%) tested positive for rabies virus.

**Figure 3:**
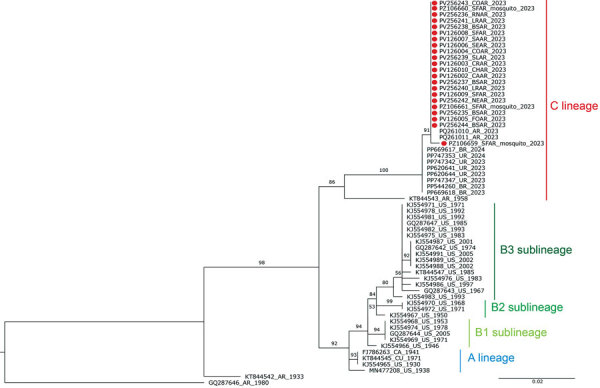
Maximum-likelihood phylogenetic tree based on partial nonstructural protein 4 gene sequences from study of epidemiologic, entomologic, and virologic findings during reemergence of western equine encephalitis virus, Argentina, 2023–2024. Analysis includes 19 newly sequenced isolates obtained from mosquitoes and equines (red dots) across a wide geographic range in Argentina. Numbers at major nodes indicate bootstrap support values based on 10,000 replicates. GenBank accession numbers are provided for reference sequences; accession numbers for all sequences from this study are provided in Tables 1 and 2 and the Appendix. Scale bar indicates number of nucleotide substitutions per site.

**Figure 4:**
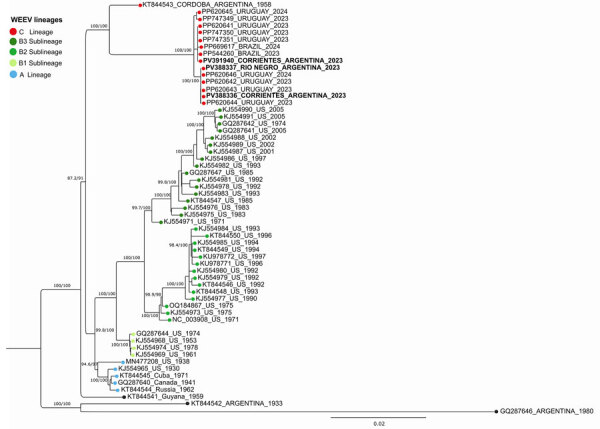
Maximum-likelihood phylogenetic tree based on complete genomes from study of epidemiologic, entomologic, and virologic findings during reemergence of western equine encephalitis virus, Argentina, 2023–2024. The tree includes 3 newly sequenced strains from Corrientes (n = 2) and Río Negro (n = 1) Provinces in Argentina, indicated in boldface, alongside reference sequences. Tip colors indicate lineages based on Bergren et al. (*24*). Numbers at major nodes indicate bootstrap support values (10,000 replicates). GenBank accession numbers are provided for reference sequences; accession numbers for all sequences are provided in Tables 1 and 2 and the Appendix . Scale bar indicates nucleotide substitutions per site.

We generated 3 novel WEEV whole genomes with >90% coverage, as well as 19 partial sequences of the nsP4 gene (Table 1). Phylogenetic analyses of both the nsP4 and whole-genome sequences indicated that the circulating WEEV strains clustered with those previously detected in Uruguay and Brazil. Moreover, they were closely related to an older viral strain isolated during the 1957 epizootic in Oncativo, Córdoba Province (GenBank accession no. KT844543.1), constituting a new lineage designated as lineage C (Figures 3, 4).

### Mosquito Collection and Alphavirus Molecular Detection

**Figure 5:**
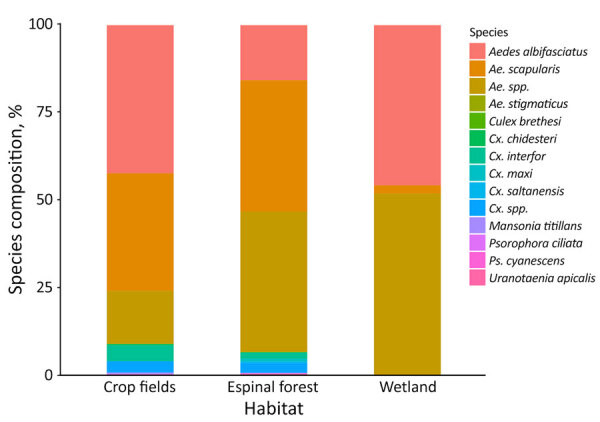
Relative abundance and species composition of mosquitoes collected from study of epidemiologic, entomologic, and virologic findings during reemergence of western equine encephalitis virus, Argentina, 2023–2024. Mosquitoes were collected by aspiration across different environments (Espinal forest patches, crop fields, and flood plains) in Santa Fe Province, Argentina. Sampling locations correspond to the sites detailed in Figure 1.

We identified 1,362 female mosquitoes belonging to 12 species from the aspirated collections. The dominant mosquito species across all analyzed environments (crop fields, Espinal forest, and wetlands) were *Ae. albifasciatus* (70.1% [955/1,362]) and *Ae. scapularis* (26.8% [365/1,362]). Less abundant species included *Cx. interfor* (1.8%), *Cx. maxi* (0.4%), *Cx. saltanensis* (0.22%), *Ur. apicalis* (0.22%), *Ma. titillans* (0.14%), and *Ae. stigmaticus*, *Cx. brethesi*, *Cx. chidesteri*, *Ps. ciliata*, and *Ps. cyanescens* (each 0.07%). The highest mosquito species diversity was observed in Espinal forest patches at Sa Pereyra, where 9 species were detected, and Marcelino Escalada, where 6 species were detected (Figure 5).

To date, we analyzed 27 mosquito pools from Arrufo (n = 1,089 mosquitoes) using the generic Alphavirus RT-PCR (Table 2). Three pools tested positive (2 from *Ae*. *albifasciatus* mosquitoes and 1 from *Ae. scapularis* mosquitoes). We sequenced those amplicons and included them in the nsP4 partial fragment phylogenetic analysis (Figure 3).

**Table 2:** Mosquito pools collected in Arrufo, San Cristóbal Department, Santa Fe Province, Argentina, during December 2023, and analyzed by generic Alphavirus reverse transcription PCR in study of reemergence of western equine encephalitis virus, Argentina, 2023–2024

Mosquito species	No. positive pools (analyzed pools)	No. mosquitoes	GenBank accession nos.
*Culex acharistus*	0 (2)	3	
*Cx. chidesteri*	0 (1)	7	
*Cx. interfor*	0 (3)	27	
*Cx. maxi*	0 (1)	10	
*Cx. saltanensis*	0 (2)	22	
*Haemagoggus spegazini*	0 (2)	12	
*Psorophora cyanescens*	0 (1)	5	
*Ps. discrucians*	0 (2)	5	
*Aedes albifasciatus*	2 (10)	972	PZ106660, PZ106661
*Ae. scapularis*	1 (3)	26	PZ106659
Total	3 (27)	1,089	

## Discussion

Argentina experienced the reemergence of WEEV after 35 years of apparent silence. The epizootic spread to neighboring countries, with >3 confirmed cases reported in Brazil and ≈1,000 in Uruguay (*14*,*15*). We identified WEEV as the etiologic agent of 1,543 cases of neurologic disease. The outbreak primarily affected equids but also involved humans, representing a substantially higher number of cases than reported during previous epizootics. Furthermore, both our study and a recent report by Vissani et al. (*13*) documented WEEV infection in sheep, indicating that other domestic species were also affected. During this epizootic, Vissani et al. (*13*) conducted histopathologic studies on fatal equine cases, demonstrating that WEEV infection was characterized by severe neutrophilic meningoencephalitis, with lesions and viral RNA most frequently detected in the internal capsule, thalamus, and brainstem.

WEEV was historically a major cause of equine and human encephalitis in both the United States and Argentina; recurrent epizootics were reported until the 1990s. Despite the absence of reported cases in the decades that followed, serologic and virologic evidence suggested that WEEV continued to circulate cryptically in South America. In 2007, a high seroprevalence (36.4%) was detected in equids on a ranch in the southern Pantanal region of Brazil (*30*). In Argentina, a 2% seroprevalence of neutralizing antibodies was reported in wild birds in Iguazú National Park (*31*), suggesting potential low-level enzootic transmission. The last documented human case before the current outbreak occurred in Uruguay in 2009 and was fatal (*5*).

On the basis of our genomic analysis, the circulating epizootic strains from the 2023–2024 outbreak are genetically similar to those recovered in Uruguay and Brazil, constituting a monophyletic lineage. This lineage is closely related to the historical Cba-87 strain, originally isolated from an equid in Oncativo, Córdoba Province, during the 1957–1958 epizootic (*8*). Those findings directly support the hypothesis that WEEV persisted silently within the region and reemerged under favorable climatic and environmental conditions, rather than being a newly introduced strain from distant endemic areas.

Entomologic and virologic surveillance of mosquito communities, conducted at the onset of the outbreak in a hotspot region, indicates that *Ae. albifasciatus* and *Ae.* *scapularis* mosquitoes were the primary vectors driving WEEV epizootic activity. The *Ae. albifasciatus* mosquito is widely distributed in Argentina and other countries in South America; its broad geographic range and ecologic plasticity highlight its epidemiologic relevance in WEEV transmission (*32*). This species is historically recognized as a competent vector during epizootics, and explosive population increases after heavy rainfall have coincided with outbreak events (*8*,*12*). As a floodwater mosquito that breeds in temporary pools in rural and periurban areas and feeds aggressively on equines, wild mammals, and humans, the *Ae. albifasciatus* mosquito likely acts as a bridge vector (*33*,*34*), enabling viral amplification and spillover from enzootic foci to equines and potentially to humans. Of note, *Ae. scapularis* mosquitoes have not typically been found infected with WEEV or observed in high abundance in affected areas. This virologic and ecologic evidence suggests its potential role as an epizootic vector in the region.

One crucial factor that could promote the resurgence of WEEV in the region might have been the discontinuation of preventive vaccination. The highest incidence was observed among rural equine populations, where vaccination coverage is often overlooked or suboptimal. Conversely, equids involved in equestrian sports maintain rigorous health protocols for international movement and were less affected. The outbreak appears to have concluded by EW 16 of 2024 in Chubut Province, likely because of intensified vaccination efforts by owners and breeders. In response, SENASA issued Resolution 115/2024, mandating vaccination against EEEV and WEEV for all equids >2 months of age and for previously unvaccinated animals.

Nevertheless, limited familiarity with the disease among veterinarians, reflecting the absence of reported cases for several decades, might have delayed early recognition of the outbreak, particularly in a setting where transmission is difficult to control without widespread vaccination. That context underscores the importance of timely technical guidance and coordinated communication. To mitigate future outbreaks, a comprehensive understanding of the ecologic, epidemiologic, and environmental drivers of WEEV emergence is urgently needed, especially in the context of global climate change. Strengthened early warning systems, improved vector and host surveillance, and proactive communication with veterinarians and equine industry stakeholders—together with targeted training to recognize the often-nonspecific neurologic manifestations of arboviral infections—are essential for timely diagnosis and effective response. The continued threat posed by emerging and reemerging arboviruses highlights the need for sustained vigilance and coordinated public and animal health strategies in Argentina and the wider region.

Appendix Additional information about epidemiologic, entomologic, and virologic findings during reemergence of western equine encephalitis virus, Argentina.
