# Supplementary material for: Epidemiologic, Entomologic, and Virologic Findings during Reemergence of Western Equine Encephalitis Virus, Argentina
Source: Emerg Infect Dis. 2026 Aug;32(8):1275–85. doi: 10.3201/eid3208.250829 (PMC13426794; doi:10.3201/eid3208.250829)
Supplement: Appendix — Additional information about epidemiologic, entomologic, and virologic findings during reemergence of western equine encephalitis virus, Argentina. [file 25-0829-Techapp-s1.pdf]

Article DOI: https://doi.org/10.3201/eid3208.250829

EID cannot ensure accessibility for supplementary materials supplied by authors. Readers who have difficulty accessing supplementary content should contact the authors for assistance.

Epidemiologic, Entomologic, and Virologic Findings during Reemergence of Western Equine Encephalitis Virus, Argentina

Appendix

Appendix Table. Metadata and GenBank accession numbers for Western Equine Encephalitis Virus isolates included in this study

GenBank accession number	Country/ year	Province/ State	Animal species	Strain	Phylogenetic tree
GQ287640	Canada/1941	Ontario	human	McMillan	Whole Genome
GQ287641	U.S./2005	California	mosquito	Imperial	Whole Genome
GQ287642	U.S./1974	California	mosquito	Kern	Partial & Whole Genome
GQ287643	U.S./1967	Montana	equine	Montana-64	Partial genome
GQ287644	U.S./1974	California	mosquito	BFS-2005	Partial & Whole Genome
GQ287646	Argentina/1980	Chaco	mosquito	AG80–646	Partial & Whole Genome
GQ287647	U.S./1985	New Mexico	mosquito	85–452NM	Partial & Whole Genome
KJ554965	U.S./1930	California	equine	unknown	Partial & Whole Genome
KJ554966	U.S./1946	California	mosquito	BFS932	Partial genome
KJ554968	U.S./1953	California	mosquito	BFS1703	Partial & Whole Genome
KJ554969	U.S./1961	California	bird	E1416–1961	Partial & Whole Genome
KJ554970	U.S./1968	California	squirrel	S8–122	Partial genome
KJ554971	U.S./1971	Texas	tortoise	TBT-235	Partial & Whole Genome
KJ554973	U.S./1975	Minnesota	mosquito	75V9291	Whole Genome
KJ554974	U.S./1978	California	mosquito	BFS09997	Partial & Whole Genome
KJ554975	U.S./1983	California	mosquito	KERN5547	Partial & Whole Genome
KJ554976	U.S./1983	California	mosquito	CHLV53	Partial & Whole Genome
KJ554977	U.S./1990	Texas	mosquito	PV02808A	Whole Genome
KJ554978	U.S./1992	California	mosquito	IMPR441	Partial & Whole Genome
KJ554979	U.S./1992	Colorado	mosquito	CO921356	Whole Genome
KJ554980	U.S./1992	Arizona	mosquito	93A38	Whole Genome
KJ554981	U.S./1992	Arizona	mosquito	93A27	Partial & Whole Genome
KJ554982	U.S./1993	Arizona	mosquito	93A30	Partial & Whole Genome
KJ554983	U.S./1993	Arizona	mosquito	93A79	Partial & Whole Genome
KJ554984	U.S./1993	California	mosquito	CNTR34	Whole Genome
KJ554985	U.S./1994	California	mosquito	Lake43	Whole Genome

GenBank accession number	Country/ year	Province/ State	Animal species	Strain	Phylogenetic tree
KJ554986	U.S./1997	Texas	mosquito	PV72102	Partial & Whole Genome
KJ554987	U.S./2001	Texas	mosquito	PV012357A	Partial & Whole Genome
KJ554988	U.S./2002	Texas	mosquito	R02PV002957B	Partial & Whole Genome
KJ554989	U.S./2002	Texas	mosquito	R02PV001807A	Partial & Whole Genome
KJ554990	U.S./2005	Texas	mosquito	R02PV003422B	Partial & Whole Genome
KJ554991	U.S./2005	Texas	mosquito	R0PV00384A	Partial & Whole Genome
KT844541	Guyana/1959	unknown	equine	TR25717	Whole Genome
KT844542	Argentina/1933	Buenos Aires	equine	Ar Enc MV	Partial & Whole Genome
KT844543	Argentina/1958	Córdoba	equine	CBA87	Partial & Whole Genome
KT844544	Russia/1962	Udmurt	mosquito	Y62–33	Whole Genome
KT844545	Cuba/1971	unknown	unknown	CU71-CPA	Partial & Whole Genome
KT844546	U.S./1992	Colorado	mosquito	CO92–1256	Whole Genome
KT844547	U.S./1985	California	mosquito	CHLV31	Partial & Whole Genome
KT844548	U.S./1993	California	mosquito	SUYA140	Whole Genome
KT844549	U.S./1994	California	mosquito	SAC 74	Whole Genome
KT844550	U.S./1996	California	mosquito	Kern 87	Whole Genome
KU978771	U.S./1996	California	turkey	97–5067	Whole Genome
KU978772	U.S./1997	California	bird	98–2435	Whole Genome
MN477208	U.S./1938	unknown	human	Fleming	Partial & Whole Genome
NC_003908	U.S./1971	Oregon		71V-1658	Whole Genome
OQ184867	U.S./1975	Colorado	human	R7973	Whole Genome
PP544260	Brazil/ 2023	Rio Grande do Sul	equine	Eq1090	Partial & Whole Genome
PP620641	Uruguay/2023	Paysandú	equine	URY_DILAVE070	Partial & Whole Genome
PP620642	Uruguay/2023	San José	equine	URY_DILAVE158	Whole Genome
PP620643	Uruguay/2023	San José	equine	URY_DILAVE198	Whole Genome
PP620644	Uruguay /2023	Paysandú	equine	URY_DILAVE218	Partial & Whole Genome
PP620645	Uruguay /2024	Rocha	equine	URY_DILAVE236	Whole Genome
PP620646	Uruguay/ 2024	San Jose	equine	RY_DILAVE255	Whole Genome
PP669617	Brazil/ 2024	Rio Grande do Sul	equine	Eq237	Partial& Whole Genome
PP669618	Brazil/2023	Rio Grande do Sul	equine	EQ1122	Partial genome
PP747342	Uruguay/2023	San Jose	equine	WEEV-UY-95	Partial genome
PP747347	Uruguay/2023	Río Negro	equine	WEEV-UY-197	Partial genome
PP747353	Uruguay/2024	San Jose	equine	WEEV-UY-248	Partial genome
PP747349	Uruguay/ 2023	Artigas	equine	WEEV-UY-199	Whole Genome
PP747350	Uruguay/ 2023	Lavalleja	equine	WEEV-UY-227	Whole Genome
PP747351	Uruguay /2023	Lavalleja	equine	WEEV-UY-228	Whole Genome
PQ261010	Argentina/2023	Unknown	equine	E.5239.23.1P	Partial genome
PQ261011	Argentina/2023	Unknown	equine	E528.23.1P	Partial genome
KJ554967	U.S./1950	Missouri	mosquito	EP-6	Partial genome
KJ554972	U.S./1971	California	mosquito	BFS3060	Partial genome
FJ786263	Canada/1941	Ontario	human	ON41-McMillan	Partial genome
